# Exploring the workplace climate and culture in relation to food environment-related factors in Norwegian kindergartens: The BRA-study

**DOI:** 10.1371/journal.pone.0225831

**Published:** 2019-12-02

**Authors:** Anne Himberg-Sundet, Anne Lene Kristiansen, Mekdes K. Gebremariam, Thomas Moser, Lene Frost Andersen, Mona Bjelland, Nanna Lien

**Affiliations:** 1 Department of Nutrition, Institute of Basic Medical Sciences, Faculty of Medicine, University of Oslo, Norway; 2 Department of Educational Science, Faculty of Humanities, Sports and Educational Science, University of South-Eastern Norway, Norway; Massachusetts Department of Public Health, UNITED STATES

## Abstract

**Background:**

Kindergartens represent an important arena for promoting vegetable intake when it is essential to establish healthy dietary behaviours early in life. To develop and implement successful interventions targeting dietary behaviours in kindergartens, a good understanding of the factors influencing their food environment and the interplay between these factors is essential. The present study aimed to explore associations between workplace climate and culture in the kindergarten setting and the staff’s food-related practices, vegetables served and the possible mediating role of staff’s food-related practices.

**Method:**

Vegetables served, staff’s food-related practices, and data on workplace climate and culture were collected using a 5-day, weighted, vegetable diary and three paper-based questionnaires. Seventy-three kindergartens in the Norwegian counties of Vestfold and Buskerud participated in the study. Spearman’s rho was used to assess the association between workplace climate and culture, and staff’s food-related practices and vegetables served. Mediation analyses were conducted to assess the mediating role of staff’s food-related practices in the relationship between workplace climate and culture and vegetables served in this setting.

**Results:**

There was one significant positive correlation between factors in the workplace climate and culture, and staff’s food-related practices and vegetables served. The staff’s food-related practices were found to mediate the association between *support from superior* and the variety of vegetables served. They also mediated the association between *commitment to the organization* and the frequency, as well as the variety, of vegetables served.

**Conclusion:**

The results identified *commitment to the organization* and *support from superior* as two important factors in the workplace climate and culture. Furthermore, these two factors seems to be important to target when developing kindergarten-based interventions aimed at increasing the variety and frequency of vegetables served as they were associated with more favourable food-related practices among staff.

## Background

Nutrition has been highlighted as one of the most critical factors for improving health and reducing the risk of non-communicable diseases [1–3], and environmental factors are emphasized as essential in their influence on diet-related behaviours [4]. Research into dietary habits in early childhood is important because it targets a crucial phase in a child’s development [1], given that dietary behaviours established early in life may carry on into adulthood [5,6]. Several interventions have aimed to increase intake of vegetables in children aged 5 years and younger [7,8]. However, only a few have been conducted in the kindergarten setting with the aim of changing the food environment. Thus, there is a lack of evidence on how to make sustainable changes in the food environment of kindergartens [8]. Workplace climate and culture in kindergartens may indirectly affect their food environment through the staff’s food-related practices, and such factors should be investigated in this setting. Workplace climate and culture consist of different psychological and social factors, which may affect the employees’ working environment [9]. These factors are distal factors including *role clarity*, *support from superior*, *support from co-workers*, *innovative climate*, *support from friends and relatives*, *commitment to the organization* and *social climate*, which are some of the relevant factors that may affect health and job performance in the workplaces [9].

In Norway, the term kindergarten’ describes an educational service for children aged 0–5 years because compulsory school starts in the year a child turns 6. Every child has a legal right to a place in a kindergarten and more than 90% of all children aged 1–5 years attend kindergartens in Norway, with the vast majority attending for 40 hours or more per week [10]. The national guidelines for food and meals in kindergartens emphasize that the kindergarten should facilitate at least three meals each day [11]. Meals are either brought from home (lunch box) or provided by the kindergarten, or a combination of the two. Few kindergartens have kitchen staff or a cook [12]. The high attendance rate makes it possible to reach many children and their families through this setting. Moreover, as kindergartens should facilitate at least three meals per day, it is essential to explore how they can provide a supportive environment for vegetable consumption.

Availability and accessibility of vegetables have been found to be important correlates for vegetable intake in school-aged children and youth [13–16], which in general are children older than the children in Norwegian kindergartens (age 1–6). In addition, a review of the evidence on how to influence younger children’s food preferences has indicated that availability, accessibility, familiarity and parental modelling are essential factors [17]. Reviews among children aged up to 18 years point to parental intake, parental modelling and parental encouragement as important factors that are positively associated with children’s fruit and vegetable consumption [14,16,18–19]. Therefore, it could be assumed that child-care staff also play an important role with regard to children’s food and vegetable intake. Three studies have examined the role of child-care staff’s modelling behaviour and shown positive associations with food-related practices and children’s food [20] and vegetable intake [21], and food acceptance [22]. In the Norwegian national guidelines for food and meals in kindergartens, it is emphasized that child-care staff should take an active role during meals, because they are important role models for the children [11].

**Fig 1:**
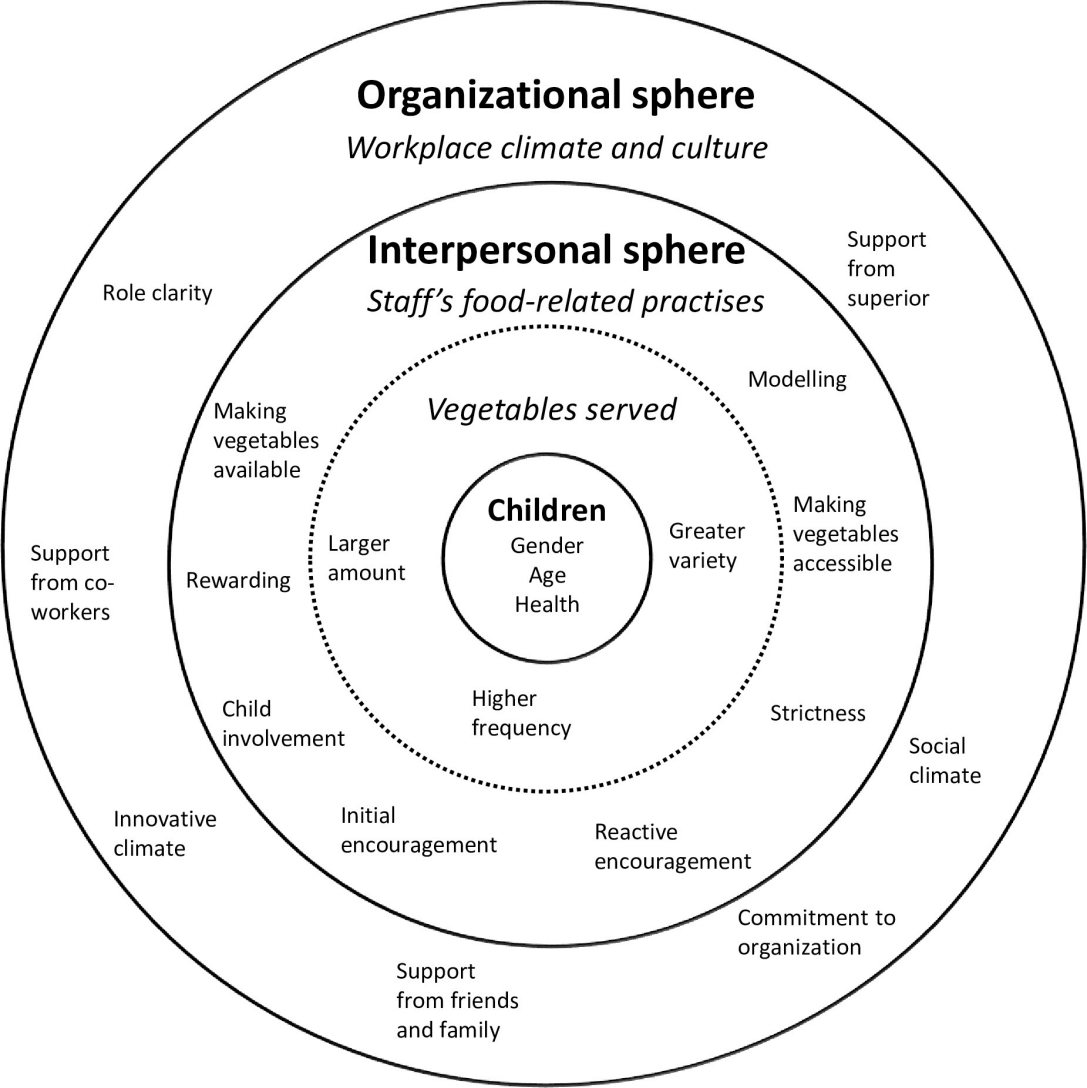
Social ecological model illustrating the different layers of factors that may affect vegetables served to children in kindergartens. (Adapted from Bartholomew et al. [24].).

More distal factors related to children’s food environment, such as workplace climate and culture in the kindergarten, may also be important in the development of successful interventions aimed at influencing the proximal factors illustrated in Fig 1. However, there is a lack of kindergarten-based studies investigating how distal factors, such as the organizational culture/climate, leadership or organizational commitment, can affect implementation [23]. It has been emphasized that it is the individuals who make up an organization who affect implementation, so there is a need to understand the social systems and structures of the organization before an intervention can be successfully implemented [24].

Following a social ecological model [24] with four layers (Fig 1), the workplace climate and culture [9] lie within the fourth layer, which makes up the organizational sphere. The distal factors of the model, such as social climate, may both limit and enable the proximal factors of the model, such as staff’s food-related practices, which in turn may limit or enable vegetables being served [24]. Illustrated in the third layer of the model (Fig 1) are *staff’s food-related practices*, which consist of *availability*, *accessibility*, *modelling*, *strictness*, *initial encouragement*, *reactive encouragement*, *child involvement* and *rewarding* [25,26]. Staff’s food-related practices are considered to be important factors in affecting children’s food environment, and are also important in terms of serving larger amounts and a greater variety of vegetables at a higher frequency to the children; this is illustrated in the second layer of Fig 1. The dotted line illustrates that staff’s food-related practices and vegetables served are both within the interpersonal sphere.

Data on how pre-school employees experience their workplace climate and culture has previously been used for comparison with child welfare workers in a Swedish study [27], which included 377 employees in pre-schools. The results for the pre-school employees were considered to be positive because the pre-school employees had high scores on *role clarity*, *support from superior*, *support from co-workers*, *social climate* and *innovative climate* [27], and such high scores may positively affect these employees’ job performance. Furthermore, a Canadian qualitative study conducted in kindergartens, with children aged 3 months up to 6 years, found that a critical determinant for organizational behaviour was the director’s strong leadership, and that leadership, health champions, organizational culture, and networking and knowledge brokering were factors that positively influenced the adoption of nutritional guidelines [28]. However, as distal factors such as workplace climate and culture might be difficult to change by intervention, it might be easier to target more proximal factors such as staff’s food-related practices.

The aim of the present study was, therefore, (1) to explore associations between workplace climate and culture in the kindergarten and staff’s food-related practices and vegetables served, and (2) to investigate the possible mediating role of staff’s food-related practices in the relationship between the workplace climate and culture and vegetables served.

## Method

### Study design and subjects

Baseline data from the BRA-study (*Barnehage* [kindergarten], *gRønnsaker* [vegetables] and *fAmilie* [family]) were used. The BRA-study is a clustered, randomized controlled, intervention study with an overall aim to improve vegetable intake among children (aged 3–5 years at baseline) through changing the food environment and dietary practices in the kindergarten and at home. In the autumn and winter 2014–2015, all 479 public and private kindergartens in Vestfold and Buskerud counties were invited to participate in the study; of these 73 kindergartens accepted (15.2% response rate). Within the 73 kindergartens, departments with children born in 2010 or 2011 were eligible for the study and 135 departments agreed to participate. Norwegian kindergartens are generally organized into different departments within each kindergarten. Each department is usually staffed by one pedagogical leader with formal responsibility for the department, in addition to two or more assistants, and consists of a wardrobe, bathroom facilities, and one or more activity/play rooms; some departments also contain some kitchen facilities (e.g. kitchen counter with sink, refrigerator).

Baseline data were collected in the spring of 2015, and a detailed description of this has previously been published [29]. In Norway, kindergarten leaders have full responsibility for the administrative tasks and ensuring the quality of the kindergarten’s pedagogical activities. Pedagogical leaders are responsible for planning and conducting the pedagogical activities within the departments, and guide the kindergarten assistants in their pedagogical work. Kindergarten assistants support the pedagogical leaders to ensure that the pedagogical activities are conducted and to care for the children. In the following, the term ‘staff’ includes employees within one kindergarten department, namely the pedagogical leader and kindergarten assistants. Data on vegetables served, staff’s food-related practices and the organization were collected using several instruments. A paper-based questionnaire, answered by the kindergarten leader, was used to assess workplace climate and culture. A paper-based questionnaire, answered by the pedagogical leaders, was used to assess the frequency and variety of vegetables served, the workplace climate and culture, and the staff’s food-related practices. A paper-based questionnaire, answered by the kindergarten assistants, was used to assess the workplace climate and culture and the staff’s food-related practices. All three groups of staff answered the same questions about the workplace climate and culture, and leaders and assistants answered the same questions about the staff’s food-related practices. The instruments differed across the three groups with regard to the number and content of additional questions, according to what was considered relevant for each group of employees.

A paper-based, 5-day weighted vegetable diary, filled in by kindergarten staff working at the participating departments, was used to assess the amount of vegetables served.

## Measurements

### Workplace climate and culture

Workplace climate and culture were assessed using scales from the validated General Nordic Questionnaire for Psychological and Social Factors at Work (QPS_Nordic_) [9]. The original QPS_Nordic_ contains 26 different scales measured by 123 items [9]. Based on consultation with one of the developers of the QPS_Nordic_, we chose to include the 8 most relevant scales, which consisted of 19 items. The scales used were: *Role clarity* (three items), *Support from superior* (three items), *Support from co-workers* (two items), *Support from friends and relatives* (two items), *Social climate* (three items), *Innovative climate* (three items) and *Commitment to the organization* (three items) (Table 1). Calculation of the scales followed the QPS_Nordic_’s user guide [9]. The response to each item was given on a 5-point scale with 5 as the highest value and 3 as a neutral midpoint. Before items were calculated, a reversed score was calculated for one item, *distrustful and superstitious*, included in the scale called *Social climate*. Measurements of kindergarten climate and culture were collected from the kindergarten leader, the pedagogical leader and the kindergarten assistants (*n* = 428); this was then aggregated to the kindergarten level (*n* = 67).

**Table 1:** Scales from QPS_Nordic_ used to measure kindergarten climate and culture: the BRA-study (*n* = 428).

Scales and items included	Cronbach’s α	Baseline Median (min., max.)
**Role clarity**^**a**^	0.83	4.67 (3.75, 5.00)
Have clear, planned goals and objectives been defined for your job?		
Do you know what your responsibilities are?		
Do you know exactly what is expected of you at work?		
**Support from superior**^**a**^	0.86	4.49 (3.05,5.00)
If necessary, can you get support and help with your work from your immediate superior?		
If necessary, is your immediate superior willing to listen to your work-related problems?		
Are your work achievements appreciated by your immediate superior?		
**Support from co-workers**^**a**^	0.78	4.60 (3.75, 5.00)
If necessary, can you get support and help with your work from your co-workers?		
If necessary, are your co-workers willing to listen to your work-related problems?		
**Support from friends and relatives**^**a**^	0.79	3.61 (1.67, 5.00)
If necessary, can you talk with your friends about your work-related problems?		
If necessary, can you talk with your spouse or any other close person about your work-related problems?		
**Social climate**^**b**^	0.67	4.07 (2.92, 4.92)
What is the climate like in your work unit? - Encouraging and supportive - Distrustful and suspicious^e^ - Relaxed and comfortable		
**Innovative climate**^**a**^	0.71	4.28 (3.33, 4.92)
Do workers take initiatives at your workplace?		
Are workers encouraged to think of ways to do things better at your workplace?		
Is there sufficient communication in your department?		
**Commitment to the organization**^**c**^	0.86	4.69 (3.08, 5.00)
To my friends I praise this organization as a great place to work		
My values are very similar to the organization’s values		
This organization really inspires me to give my very best job performance		

^a^Precoded answer categories: *very seldom or never*, *quite seldom*, *sometimes*, *quite often*, *very often or always*.

^b^Precoded answer categories: *very little or not at all*, *quite little*, *somewhat*, *quite a lot*, *a great deal*.

^c^Precoded answer categories: *disagree totally*, *disagree to some extent*, *indifferent*, *agree to some extent*, *agree totally*.

^e^Reversed score.

### Kindergarten staff’s food-related practices

Using a previous paper from the BRA-study [26], we used the score labelled ‘staff’s food-related practices’, which consisted of eight factors containing three to five items each. A detailed description of how the factors were extracted has been published elsewhere [25]. The description of how this score was calculated has also been published previously [Himberg-Sundet et al, accepted]. The eight factors were: *modelling* (five items), *initial encouragement* (five items), *child involvement* (four items), *reactive encouragement* (three items), *strictness* (three items), *rewarding* (three items), *accessibility* (four items) and *availability* (three items) [25]. Measurements of staff’s food-related practices were collected from assistants and leaders (*n* = 373), and then aggregated to the kindergarten level (*n* = 67).

### Frequency and variety of vegetables served

A detailed description of data collection in terms of the measurements of frequency, variety and amount of vegetables served has been published previously [26]. The frequency of served vegetables for breakfast, lunch and the afternoon meal was assessed through three separate questions: ‘How often does your department offer vegetables for breakfast/lunch/afternoon meal?’ The response alternatives were on a 7-point scale ranging from ‘5 days a week’ to ‘1–3 times per month’ and ‘never’. The variety of vegetables served for lunch and the afternoon meal was also assessed through three separate questions: ‘How often does your department offer these vegetables for breakfast/lunch/afternoon meal?’ Twelve kinds of vegetables were given as response alternatives, using the same 7-point scale mentioned above. From these data the monthly variety of vegetables served was calculated, by first recoding the 7-point scale: 5 days a week = 5, 4 days a week = 4, 3 days a week = 3, 2 days a week = 2, 1 day a week = 1, 1–3 times a month = 0.5, never = 0. If a vegetable had been served (regardless of how many times per week/month) a value of 1 was given, whereas, if a vegetable had not been served, a value of 0 was given. Then the variety of vegetables served for breakfast, lunch and the afternoon meal was calculated. Measurements of frequency and variety of vegetables served were collected from 110 pedagogical leaders, and then aggregated to the kindergarten level (*n* = 67).

### Amount of vegetables served

A detailed description of the 5-day weighted vegetable diary used to assess the amount of vegetables served has been published previously [29]. Briefly, the kindergarten staff were instructed to weigh vegetables served for 5 consecutive days before each meal, and to note the number of children and adults present. This provided a measure of the amount of vegetables, in grams, served per person per day. Measurements of the amount of vegetables served were collected from 109 kindergarten departments, and then aggregated to the kindergarten level (*n* = 67).

### Data analysis

All analyses were conducted after aggregating the data to the kindergarten level. To base all analyses on the same sample, only kindergartens with complete data about the workplace climate and culture, the staff’s food-related practices and vegetables served were included (*n* = 67). Median, minimum and maximum scores were calculated and presented for the scales and the single items used from the scale. As some of the measures were slightly skewed, we chose to use median scores. Cronbach’s alpha was calculated to assess the internal consistency of the items included in the scales. As some of the scales and items measuring workplace climate and culture were not normally distributed, Spearman’s rho was used in the calculation of bivariate correlations between climate and culture in the kindergarten and staff’s food-related practices and vegetables served.

**Fig 2:**
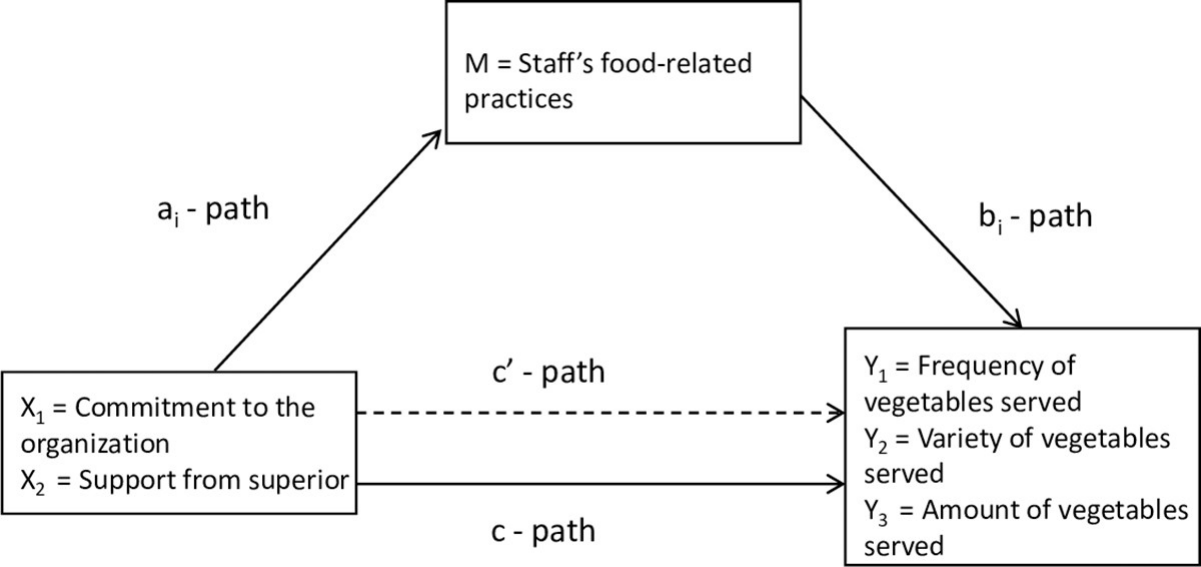
Simple mediation models for the association between (1) *commitment to the organization* and (2) *support from superior*, and the frequency, variety and amount of vegetables served.

Based on the logic model for the intervention, the secondary outcomes for the BRA-study [26], and Cohen’s guidelines for interpretation of correlation coefficients by the inclusion of correlation >0.3, six mediation models were used (Fig 2). These six mediation models assessed the mediating role of staff’s food-related practices, exploring the role of staff’s food-related practices in the association across: (1) *commitment to the organization* and frequency of vegetables served, (2) *commitment to the organization* and variety of vegetables served, (3) *commitment to the organization* and amount of vegetables served, (4) *support from superior* and frequency of vegetables served, (5) *support from superior* and variety of vegetables served and (6) *support from superior* and amount of vegetables served. Mediation analyses were conducted using the PROCESS SPSS macro provided by Hayes [30], and non-standardized beta coefficients are presented. For small sample sizes, it is recommended that the bootstrapping method be used [30]. Furthermore, the PROCESS macro provides percentile bootstrap confidence intervals that have been shown to be more robust with regard to small sample sizes [30]. Therefore, 5000 bootstrap re-samples, with 95% bias-corrected confidence intervals for the indirect effect, were conducted [30]. The assumptions of linearity, normality and homoscedasticity were investigated and considered acceptable. The residuals for the mediation models were inspected and confirmed as normally distributed. Fig 2 shows the steps followed in the mediation analyses: (1) the c-path measures the total effect of climate and culture in the kindergarten (*commitment to the organization* and *support from superior*) on vegetables served (frequency, variety and amount). (2) The a-path measures the relationship of climate and culture in the kindergarten (*commitment to the organization* and *support from superior*) with the possible mediator staff’s food-related practices, in all six models. (3) The b-path measures the relationship between the mediator staff’s food-related practices and vegetables served (frequency, variety and amount). (4) The c′-path measures the direct effect of climate and culture in the kindergarten on vegetables served (frequency, variety and amount) when adjusting for the staff’s food-related practices. Eventually, the indirect effect of the possible mediating variable (a-path × b-path) was investigated for each of the models [31,32]. Statistical analyses were conducted using SPSS^®^ version 24.0.

## Results

The descriptive statistics of the kindergartens at baseline has been previously published [29]. The baseline mean score (standard deviation [SD]) for the variety of vegetables served was 7.7 (3) times per month, the frequency of vegetables served had a mean score of 7.2 (3.7) times per week, whereas the mean score for the amount of vegetables served was 44 (24) grams per person per day [26]. The mean score for staff’s food-related practices at baseline was 4.0. (0.5); more descriptive statistics for this score can be found elsewhere [26]. The baseline median scores for the different scales from the QPS_Nordic_ are presented in Table 1. The internal consistency of the scales (Cronbach’s alpha) varied between 0.67 and 0.86 (Table 1), which is comparable to the results from the original QPS_Nordic_ questionnaire [9] of 0.71–0.83.

Correlations between the scales that measure the workplace climate and culture and staff’s food-related practices and vegetables served are presented in Table 2. *Commitment to the organization* (*r*_*s*_ = 0.39) was significantly positively correlated with the staff’s food-related practices. *Support from superior* (*r*_*s*_ = 0.33) was positively correlated with the staff’s food-related practices. Further, *support from superior* (*r*_*s*_ = 0.33), *support from co-workers* (*r*_*s*_ = 0.31) and *commitment to the organization* (r = 0.32) were positively correlated with the frequency of vegetables served.

**Table 2:** Correlation of QPS_Nordic_ scales and staff’s food-related practices and vegetables served (*n* = 67).

Measure	Staff’s food-related practices Spearman’s rho (p)	Frequency^a^ of vegetables served Spearman’s rho (p)	Variety^b^ of vegetables served Spearman’s rho (p)	Amount^c^ of vegetables served Spearman’s rho (p)
Role clarity	0.18 (0.136)	0.21 (0.088)	0.05 (0.711)	‒0.07 (0.564)
Support from superior	0.33 (0.006)	0.33 (0.006)	0.03 (0.833)	0.20 (0.104)
Support from co-workers	0.23 (0.058)	0.31 (0.011)	0.13 (0.301)	0.11 (0.355)
Support from friends and relatives	‒0.00 (0.984)	0.08 (0.522)	0.05 (0.665)	0.08 (0.524)
Social climate	0.17 (0.169)	0.26 (0.034)	0.19 (0.132)	‒0.07 (0.564)
Innovative climate	0.18 (0.137)	0.26 (0.031)	0.20 (0.109)	‒0.08 (0.951)
Commitment to the organization	0.39 (0.001)*	0.32 (0.008)	0.23 (0.058)	0.12 (0.333)

Statistical significance at *p <0.002, set using Bonferroni’s correction.

^a^Frequency of vegetables served over a week.

^b^Variety of vegetables served over a month.

^c^Amount of vegetables served per person per day.

Even though only one significant correlation was found using Bonferroni’s correction (*p* = 0.002), we decided to explore factors with correlations >0.3 in mediation analysis. As there can be mediation without a significant main effect [30], we proceeded with mediation analysis. Single mediation analyses revealed no significant effect of *commitment to the organization* on the frequency, variety and amount of vegetables served (c-path and c′-path) (Table 3). A significant total and direct effect (*p* <0.05) was found only for *support from superior* on the frequency of vegetables served (c = 2.37, c′ = 1.80). A significant mediation effect (*p* <0.05) of the staff’s food-related practices was found on the relationship between *support from superior* and variety of vegetables served (ab = 0.66, confidence interval [CI] = 0.10, 1.32) (a-path × b-path) (Table 3). In addition, a significant mediation effect (*p* <0.05) of the staff’s food-related practices was found for the relationship between *commitment to the organization* and frequency of vegetables served (ab = 0.77, CI = 0.05, 1.48) (a-path × b-path) and variety of vegetables served (ab = 0.71, CI = 0.01, 1.44) (a-path × b-path).

**Table 3:** Mediation effect of the staff’s food-related practices in the association between climate and culture in the kindergarten and vegetables served (*n* = 67).

Measurement	c-path (SE)	c′-path (SE)	a-path (SE)	b-path (SE)	ab^a^	95% CI
**Outcome: frequency of vegetables served (per week)**						
Commitment to the organization	1.68* (0.82)	0.91 (0.85)	0.24** (0.08)	3.24** (1.29)	0.77	0.05, 1.48
Support from superior	2.37** (0.80)	1.80* (0.81)	0.20** (0.08)	2.91* (1.23)	0.58	‒0.03, 1.16
**Outcome: variety of vegetables served (per month)**						
Commitment to the organization	1.03 (0.67)	0.33 (0.68)	0.24** (0.08)	2.98** (1.03)	0.71	0.10, 1.32
Support from superior	0.27 (0.68)	‒0.39 (0.70)	0.20** (0.08)	3.33** (1.02)	0.66	0.10, 1.32
**Outcome: amount of vegetables served (grams per person per day)**						
Commitment to the organization	7.88 (6.70)	3.67 (7.10)	0.24** (0.08)	17.75 (10.77)	4.21	‒1.05, 9.76
Support from superior	10.69 (6.62)	7.45 (6.87)	0.20** (0.08)	16.32 (10.46)	3.24	‒0.78, 8.54

CI, confidence interval; SE, standard error.

Statistical significance at *p<0.05 and **p<0.01.

^a^Indirect effect calculated as a-path × b-path

## Discussion

The present study has explored associations between workplace climate and culture in the kindergarten and the staff’s food-related practices, and the frequency, variety and amount of vegetables served. It also explored the mediating role of the staff’s food-related practices in the relationship between workplace climate and culture in the kindergarten and vegetables served. Results showed one significantly positive correlation (*p* = 0.001) between *commitment to the organization* and the staff’s food-related practices. The results showed moderate positive correlations between *commitment to the organization* and frequency of vegetables served, and *support from superior* with the staff’s food-related practices and frequency of vegetables served, although the associations did not reach statistical significance at the *p* value set after Bonferroni’s correction. The staff’s food-related practices were found to mediate the association between *support from superior* and the variety of vegetables served. Staff’s food-related practices also mediated the association between *commitment to the organization* and the frequency, as well as the variety, of vegetables served.

### Workplace climate and culture in kindergartens

Previously conducted studies on psychological and social factors in the kindergarten setting have mostly focused on the burden of stress in this field of work [33,34]; other distal factors have not previously been explored in this context. Compared with results from a study of social workers in Sweden, which included pre-school teachers as a comparison group [27], our results revealed that the kindergartens included in the present study had a favourable workplace climate and culture. This may be partly due to the kindergartens participating in the present study having a more engaged group of employees.

### Associations between workplace climate and culture, staff’s food-related practices and vegetables served

The results showed only one significantly positive association between factors in the workplace climate and culture and the staff’s food-related practices and vegetables served. Even though they were not significant, the results showed moderate positive correlations between *commitment to the organization* and frequency of vegetables served, and *support from superior* with staff’s food-related practices and frequency of vegetables served. To the authors’ knowledge, no studies have been conducted on associations between these specific organizational factors in the kindergarten and the staff’s food-related practices, and vegetables served. Several school-based studies have shown how some of these distal factors might affect job performance [35–38], pointing to organizational commitment promoting organizational effectiveness within the educational system [35,36] and job performance among teachers [38]. Thus, the positive correlation between *commitment to the organization* and the staff’s food-related practices may be a result of the kindergarten employees’ high degree of commitment to the workplace. Also, a servant leadership style (e.g. praise and support, listening to people and caring about their needs) may be useful in enhancing a teacher’s job satisfaction [39], which in turn improves effective work [40]. As a servant leadership style has several similarities to social support (listening to, praise and support others), this may explain why support from superior correlates positively with the staff’s food-related practices, and frequency of vegetables served, because this leadership style may improve effective work and enhance teachers’ job satisfaction.

Several of the factors measuring workplace climate and culture in the kindergarten were not associated with the staff’s food-related practices or vegetables served. The lack of associations for *support from friends and relatives* may be explained by the confidentiality agreement that kindergarten employees must sign before starting to work in the kindergarten, and thus could lead to kindergarten employees not seeking support from friends and relatives with regard to work. This was also mentioned by a number of respondents.

### The mediating role of staff’s food-related practices on the relationship between workplace climate and culture and vegetables served

Although a direct relationship was not observed, the mediation analyses showed significant mediation effects of the staff’s food-related practices on the relationship between *commitment to the organization* and the frequency and variety of vegetables served, and between *support from superior* and the variety of vegetables served. These results from the mediation analyses indicate that factors in the workplace climate and culture may affect vegetables served through staff’s food-related practices. This is supported by a Canadian study [28] which found that both leadership and organizational culture might be important factors for the implementation of nutritional guidelines in kindergartens. Based on this knowledge one should address factors in the workplace climate and culture in future interventions to facilitate change in staff’s food-related practices, since previous results from the BRA-study have found that targeting the staff’s food-related practices directly through training did not result in change [26]. The mediation results, points to the importance of leaders being supportive during the intervention, by listening to, praising and supporting the kindergarten employees. In addition, a supportive leader might engage the employees early in the process of the development of an intervention, to ensure commitment throughout the implementation. However, as *commitment to the organization* and *support from superior* showed relatively high scores for the kindergartens participating in the BRA-study, an increase in this score might be challenging.

As these mechanisms between distal and more proximal factors in the kindergarten environment seem complex, further studies are needed to explore how these factors interact or are mediated by each other to develop more successful interventions in this setting.

### Strengths and limitations

The present study has a number of strengths. First, few studies have investigated factors in the kindergarten environment. Second, this was the first study to highlight factors in the kindergarten workplace climate and culture, and that these factors may be of importance in developing interventions aimed at increasing the variety and amount of vegetables served in this setting. To our knowledge, questions from the QPS_Nordic_ have never previously been used in a kindergarten setting. The present study revealed that the internal consistency of the scales, as measured by Cronbach’s alpha, was comparable with the results from the original QPS_Nordic_ questionnaire [9]. Even though the response rate was low, the kindergartens included in the BRA-study did not appear to differ from the other kindergartens in the two counties from which we recruited participants [26].

The present study also has a number of limitations. All questionnaires were paper based, including enquiries about age and gender, and the department in which the employees worked. As mentioned by a few responders, if questionnaires were compiled before being sent from the kindergarten in one prepaid envelope, this information could make it possible for other employees within the same kindergarten to identify the respondents. This perception of a lack of anonymity may have affected their answers–especially with regard to the questions used from the QPS_Nordic_. Responses to each item in the QPS_Nordic_ were given on a 5-point scale, so this might have contributed to a ceiling effect. In addition, the present study was conducted using a small sample size of kindergartens, which limits the power of the mediation analyses, and the results should be interpreted with caution.

## Conclusion

The present study showed a significantly positive correlation between *commitment to the organization* and the staff’s food-related practices. The staff’s food-related practices mediated the association between *commitment to the organization* on frequency and variety of vegetables served, and the association between *support from superior* and the variety of vegetables served. These results indicate that *commitment to organization* and *support from superior* as factors in workplace climate and culture seems to be important to target when developing kindergarten-based interventions aimed at increasing the frequency and variety of vegetables served as they related to more favourable food-related practices among staff.

## Supporting information

S1 Dataset (SAV)
